# Surface Charge Effects for the Hydrogen Evolution Reaction on Pt(111) Using a Modified Grand-Canonical Potential Kinetics Method

**DOI:** 10.3390/molecules29081813

**Published:** 2024-04-17

**Authors:** Shaoyu Kong, Min Ouyang, Yi An, Wei Cao, Xiaobo Chen

**Affiliations:** Guangzhou Key Laboratory of Vacuum Coating Technologies and New Energy Materials, Guangdong Provincial Engineering Technology Research Center of Vacuum Coating Technologies and New Energy Materials, Department of Physics, College of Physics & Optoelectronic Engineering, Jinan University, Guangzhou 510632, China; 13660941493@163.com (S.K.); anode_min@126.com (M.O.); edithan@stu2019.jnu.edu.cn (Y.A.); c18770704136@126.com (W.C.)

**Keywords:** Pt(111), hydrogen adsorption energy, hydrogen evolution reaction, charge effect, modified grand-canonical potential kinetics method

## Abstract

Surface charges of catalysts have important influences on the thermodynamics and kinetics of electrochemical reactions. Herein, we develop a modified version of the grand-canonical potential kinetics (GCP-K) method based on density functional theory (DFT) calculations to explore the effect of surface charges on reaction thermodynamics and kinetics. Using the hydrogen evolution reaction (HER) on the Pt(111) surface as an example, we show how to track the change of surface charge in a reaction and how to analyze its influence on the kinetics. Grand-canonical calculations demonstrate that the optimum hydrogen adsorption energy on Pt under the standard hydrogen electrode condition (SHE) is around −0.2 eV, rather than 0 eV established under the canonical ensemble, due to the high density of surface negative charges. By separating the surface charges that can freely exchange with the external electron reservoir, we obtain a Tafel barrier that is in good agreement with the experimental result. During the Tafel reaction, the net electron inflow into the catalyst leads to a stabilization of canonical energy and a destabilization of the charge-dependent grand-canonical component. This study provides a practical method for obtaining accurate grand-canonical reaction energetics and analyzing the surface charge induced changes.

## 1. Introduction

Hydrogen electrocatalysis reactions, including the hydrogen evolution reaction (HER) and hydrogen oxidation reaction (HOR), have been the cornerstone of the hydrogen economy and carbon neutralization [1,2,3]. Among all of the HER catalysts, platinum (Pt) has the best HER/HOR performance and thus has been the only one option available for commercial applications [4,5,6]. Although there have been extensive studies on the reaction thermodynamics and kinetics on Pt [6,7,8,9,10], using both experimental and computational methods, nearly all of the existing theoretical calculations are based on the electronic canonical ensemble (CE), which assumes a charge neutral reaction condition and ignores the electrochemical surface charges. Under electrochemical conditions, however, electrons flow into or out from a catalyst so that the electronic chemical potential keeps in balance with the external voltage [11]. This induces a dynamical change of the surface charge as the reaction is proceeding, which may generate substantial impacts on the reaction thermodynamics and kinetics. This effect cannot be captured under the electronic CE.

The surface charge effect has been attracting increasing attention in recent years. Han and coworkers found that both the reaction energy and barrier of the HER on S vacancies of MoS_2_ decrease when the negative charges on the catalyst surface increase [12]. However, their calculations are based on the electronic CE, which impedes an accurate description of the charge dependence. Liu et al. used the grand-canonical DFT package JDFTx to study the HER reaction thermodynamics of a series of catalysts, and found that the grand-canonical hydrogen adsorption energies are dramatically different from the canonical counterparts due to the impact of surface charges [11]. Meanwhile, Goddard et al. proposed a grand-canonical potential kinetics (GCP-K) model, which uses a quadratic GCP constructed from a set of canonical calculations to locate the grand-canonical geometry *R* and surface charge *n* of a catalyst during an electrochemical reaction [13]. This model provides a theoretical fundamental to explore practical approaches to address the effect of surface charge on reaction kinetics. However, it fails to quantitatively predict the grand-canonical reaction barrier and Tafel slope because it uses the total charge of an electrochemical double layer (EDL) system to represent the catalyst surface charge. Though different schemes have also been developed to implement the grand-canonical ensemble in the DFT framework [14,15], it remains a challenge to accurately predict reaction kinetics properties, hitherto. Recently, Chen and coworkers developed a grand-canonical iteration method based on DFT calculations to accurately locate the transition state and, meanwhile, to track the continuous change of surface charge in a reaction [16]. This method enables a direct optimization of grand-canonical energy with respect to *n* and *R* for both stable states and transition states without knowing the accurate analytical expression of the GCP. It can also separate the stabilization and the destabilization effects induced by surface charge variation, facilitating the analysis of the reaction mechanism beyond the traditional Butler–Volmer picture.

In this study, we propose a modified version of the GCP-K method, which is capable of obtaining reaction barriers that are in good agreement with the experimental results. This method separates the surface charge on the catalyst side from the total charge of an EDL system, making it possible to obtain accurate results of reaction energetics. Using the Pt(111) system as an example, we show that surface charges have substantial influences on both the reaction thermodynamics and kinetics. Hydrogen adsorption on Pt(111) under the SHE condition is much stronger than what we have assumed due to the high density of surface negative charges. We also demonstrate that surface charges can change the reaction barrier even for the Tafel reaction by affecting the charge-dependent grand-canonical energy term. This study provides a practical method for obtaining accurate grand-canonical reaction energetics and analyzing the surface charge induced changes.

## 2. Results and Discussions

### 2.1. Structure Models

**Figure 1:**
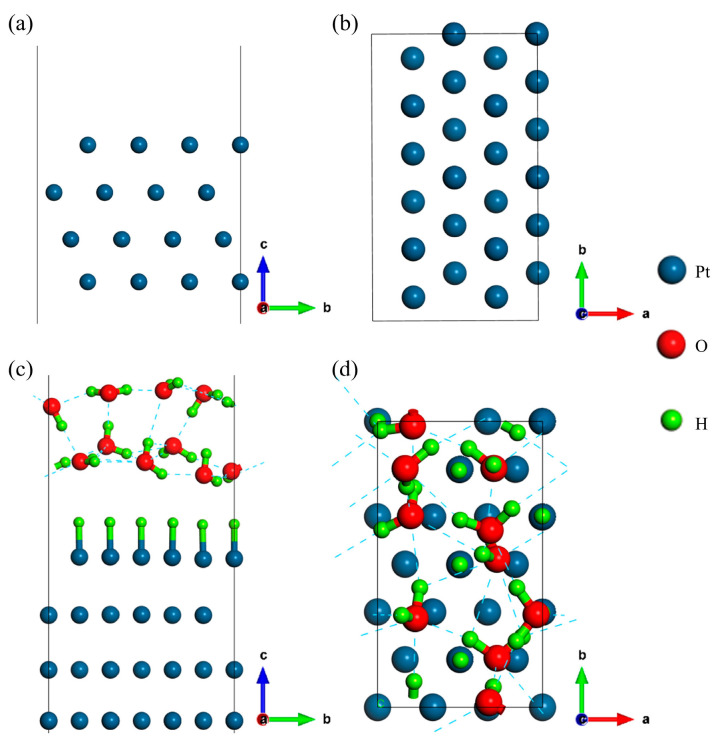
Structural models of the Pt(111) surface and the Pt(111)/H_2_O interface. (**a**) Side, and (**b**) top views of Pt(111); (**c**) side, and (**d**) top views of the Pt(111)/H_2_O interface under the SHE condition. The vectors a, b, and c represent the x, y, and z direction in the Cartesian space, respectively.

A bare Pt(111) surface was simulated using a rectangular slab model consisting of four layers of Pt atoms, as shown in Figure 1a,b. The atoms at the bottom two layers were fixed at their bulk positions, whereas the top two layers were allowed to relax. There are eight on-top sites available for hydrogen adsorption. A thick vacuum region of 20 Å was used to separate the neighboring structural images. To model the Pt(111)/H_2_O interface, 10 water molecules were added onto the surface to construct an EDL. Hydrogen atoms were added into or removed from the solution to obtain different proton concentrations. Different water networks could be obtained by sampling the conformational space using *ab-initio* molecular dynamics simulations (AIMD). According to a previous Pourbaix diagram calculation [17], the Pt(111) surface has a 100% hydrogen coverage and the proton concentration is 0% within the Helmholtz layer under the standard hydrogen electrode (SHE) condition. Therefore, we constructed a series of EDL structures with this characteristic, but with different water networks. Then, we selected the lowest energy structure around the equilibrium potential as the thermodynamically stable interfacial structure in acidic environment to study the reaction thermodynamics and kinetics. In fact, the surface charge effect exists for all electrochemical conditions, including both acidic and basic cases. The magnitude of this effect is not determined explicitly by the pH of solution. Instead, it relies on the change of surface charge during a reaction in terms of Equation (8). For simulating the basic condition, a separate Pourbaix diagram for the interface including alkali metal ions in solution should be established carefully, which is computationally expensive and beyond the range of this work.

### 2.2. Hydrogen Adsorption Energies

**Figure 2:**
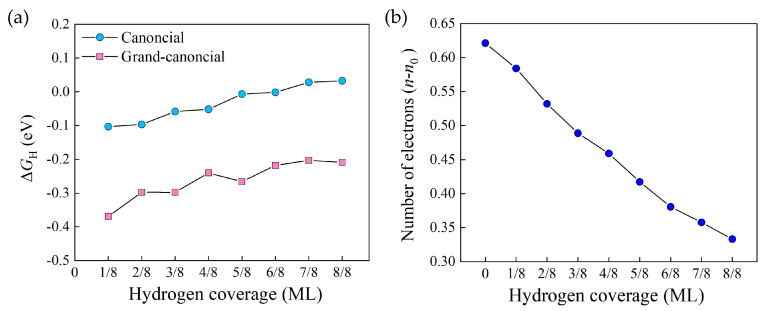
(**a**) H adsorption energy Δ*G*_H_ as a function of H Coverage on the Pt(111) Surface under the SHE potential. Both canonical and grand-canonical results are shown. (**b**) Net electron number *n*_total_ = *n* − *n*_0_ as a function of H Coverage. *n* is the total number of electrons in the system and *n*_0_ is the electron number at the electroneutral state. ML means one monolayer of adsorbed H atoms.

H adsorption energy Δ*G*_H_ has long been used as an effective descriptor to evaluate the HER activity [18]. Quite a lot of canonical DFT calculations have indicated that H adsorption on the Pt surface is thermodynamically neutral, i.e., Δ*G*_H,CE_ ≈ 0 eV, under the SHE condition, which accounts for the state-of-the-art HER activity that was experimentally observed. In Figure 2a, we plot the calculated Δ*G*_H_ as a function of H coverage by both canonical and grand-canonical ensembles. For the latter case, the potential *U* is fixed at 0 V, and the variation of the total electron number *n*_total_ with H coverage is plotted in Figure 2b.

As expected, the canonical Δ*G*_H, CE_ is close to zero for the whole range of H coverage, consistent with previous calculations [8,18]. For the high coverage region around one, which is the case of the SHE condition in acidic environment [8,17], Δ*G*_H,CE_ is slightly positive, indicating that H desorption should be easy. For both ensembles, Δ*G*_H_ increases as H coverage increases. Interestingly, Δ*G*_H,GCE_ is much more negative than Δ*G*_H,CE_, indicating that hydrogen adsorption on Pt is much stronger than what we have assumed if the potential is kept fixed during calculation. It is of note that the volcano plot established in the literature based on canonical DFT calculations locates the optimum Δ*G*_H_ to 0 eV [18,19]. Our calculation challenges this traditional belief and suggests that the optimum Δ*G*_H_ should be moved to a more negative position of ca. −0.2 eV. The reason for this change could be ascribed to the surface charge effect, as revealed in Figure 2b. For the low coverage region, the surface features a high density of negative charges (a large *n*_total_), and as H coverage increases, *n*_total_ decreases gradually. This high density of negative charges induces an enhanced adsorption of H atoms on the Pt surface. This effect is totally ignored in canonical ensemble calculations.

Although this result is difficult to be experimentally validated, it can be rationalized in an indirect manner. The 100% H-covered Pt(111) surface has ca. 0.33 electrons under the grand-canonical ensemble (Figure 2b). Using the method of our previous work [20], we calculate the proton affinities for the electroneutral and the negatively charged surfaces with 100% H coverage, which are 0.559 and 0.573 eV, respectively. The larger proton affinity for the latter demonstrates that hydrogen adsorption is indeed enhanced when the surface is negatively charged.

### 2.3. Reaction Kinetics

Using the EDL structure established in Figure 1c, which is suggested by a previous Pourbaix diagram calculation [17], we can move to calculate the reaction kinetics using both the canonical and the modified grand-canonical method. In acidic environment, H atoms adsorb on the Pt surface underpotentially [8,21]. Therefore, one only needs to consider H desorption pathways. Because previous studies have demonstrated that the Tafel route is superior to the Heyrovsky route [8,22], we take the Tafel route as an example to study the surface charge effect.

**Figure 3:**
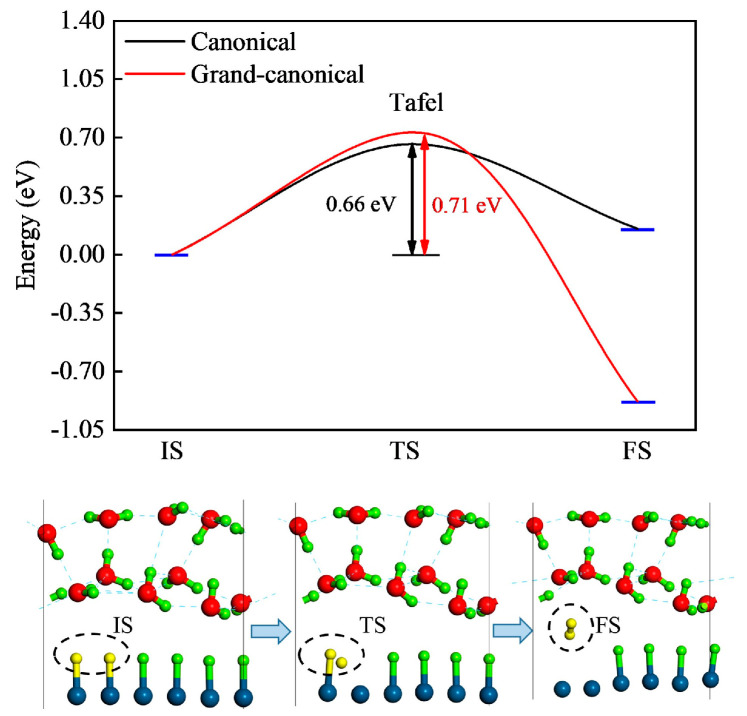
Tafel pathway for hydrogen desorption in acid. Both canonical and grand-canonical results are shown. Yellow balls in each interfacial structure represents the hydrogen atoms taking part in the reaction.

As shown in Figure 3, the Tafel reaction takes place when two adsorbed H atoms on the surface combine together to form an H_2_ molecule. The canonical free energy barrier is calculated to be 0.66 eV, with all of the correction terms summarized in Table S1. The experimental exchange current density *j*_0_ of Pt is 3.162 mA·cm^−2^ [23]. According to the relationship between Turnover Frequency (TOF) and *j*_0_,

(1) $$TOF=\frac{j_{0}}{2qN}$$

one can derive the reaction rate on a single active site TOF = 20.20 s^−1^·site^·−1^. Based on the transition state equation,

(2) $$k\left(U\right)=\frac{k_{B}T}{h}exp(-\frac{{\Delta G}^{‡}\left(U\right)}{k_{B}T})$$

TOF can be transformed to the reaction barrier Δ*G*^‡^ = 0.69 eV. Obviously, the canonical Tafel barrier is in good agreement with the experimental result.

**Figure 4:**
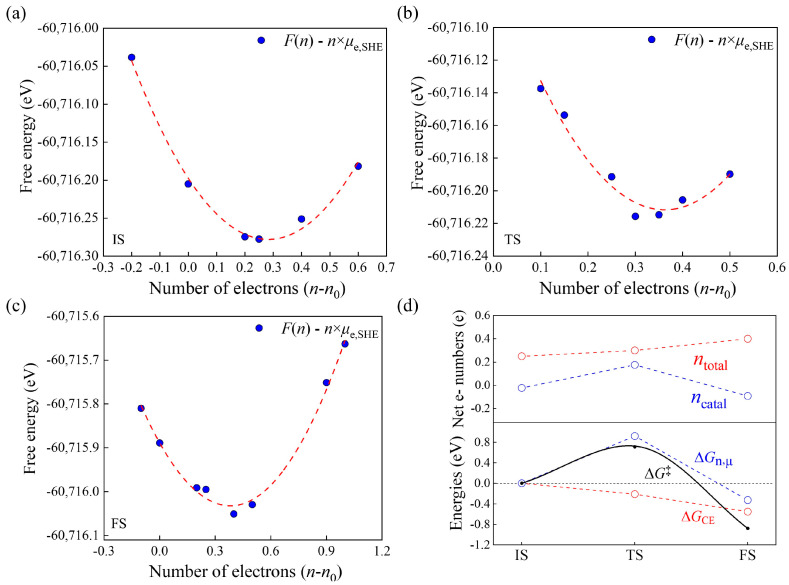
Net electron number *n*_total_ = *n* − *n*_0_ as a function of grand-canonical free energy in IS (**a**); TS (**b**) and FS (**c**) of Tafel. *n* is the total number of electrons in the system and *n*_0_ is the electron number at the electroneutral state. The red curve is the polynomial fitting curve. (**d**) Net electron numbers and energies for the Tafel reactions. Upper panel: Net electron number of the EDL system (*n*_total_) and that on the catalyst side (*n*_catal_) along the reaction routes. Lower panel: Relative changes in reaction free energy Δ*G*^‡^, canonical energy Δ*G*_CE_ and the grand-canonical part Δ*G*_n,μ_ = −Δ*n*_catal_ × *μ*_e,SHE_ along the routes of Tafel reactions.

We then evaluate the grand-canonical barrier using the modified GCP-K method introduced in Section 3.4. For the initial state (IS) and final state (FS) of the reaction, the calculation takes two steps. First, we use VASP to relax the EDL structures with different electron numbers *n* and JDFTx to obtain the corresponding canonical JSSE *F*(*n*). Then, we use a grand-canonical potential *G*(*n*, *U* = 0 V) = *F*(*n*) − *n* × *μ*_e,SHE_ to perform a quadratic fitting for the *F*(*n*)~*n* relationship at *U* = 0 V [13]. For an electrode potential *U* other than 0 V, one can perform a quadratic fitting for the grand-canonical potential *G*(*n*, *U*_SHE_) = *F*(*n*) − *n* × (*μ*_e,SHE_ − e*U*_SHE_) at the potential *U* to obtain the corresponding barrier. The results are shown in Figure 4a,c. The minimums of the curves determine the net electron numbers *n*_total_ = *n* − *n*₀, grand-canonical energies *G*_IS/FS,GCE_ and geometry structures for the IS and FS. Calculation of the grand-canonical transition state (TS) follows a similar method. The difference is that the CI-NEB method is used in the first step as the optimizer to locate the TS structures with different *n*. The result of the quadratic fitting is exhibited in Figure 4b.

From the minimums in Figure 4a–c, we derive *G*_IS,GCE_ = −60,716.28 eV, *G*_TS,GCE_ = −60,716.22 eV and *G*_FS,GCE_ = −60,716.05 eV. One can also obtain *n*_IS, total_ = 0.25, *n*_TS,total_ = 0.30 and *n*_FS,total_ = 0.40. If we use Equation (7), i.e., the original GCP-K method, to calculate reaction barrier, then Δ*G*^‡^ = 0.06 eV is obtained, which is obviously too small compared to the experimental barrier of 0.69 eV [23]. This is because Equation (7) includes the contribution of *n*_total_, and thus overestimates the surface charge and Δ*G*^‡^.

We now use Equation (8) to evaluate the reaction barrier. First of all, one needs to determine *n*_catal_ for the three states. By Bader charge analysis, we obtain the total electron number *n* for all of the water molecules at the IS and TS of the Tafel reaction, as shown in Table 1. From the IS to the TS, the catalyst obtains 0.05 electrons (*n*_total_, Figure 4d). The four water molecules highlighted by “*” in Table 1 also obtain electrons, which means that they behave the same way as the catalyst in exchanging electrons with the external electron reservoir. Therefore, they should be treated as the catalyst side when calculating *n*_catal_. The reason why one should do this is that electrons from the external circuit may tunnel through the interface and electrify some adjacent water molecules in the Helmholtz layer. We obtain *n*_IS,catal_ = −0.02, *n*_TS,catal_ = 0.18 and *n*_FS,catal_ = −0.09, which means that Δ*n*_catal_ = 0.20 when moving from the IS to the TS. Using this value in Equation (8), we then derive the grand-canonical barrier Δ*G*^‡^ = 0.71 eV (Figure 3), which is in good agreement with the experimental value of 0.69 eV. In terms of Equation (2), one can estimate that the corresponding reaction rate is 6.10 s^−1^·site^−1^. In fact, a similar enhanced agreement has also been reported for S vacancies on MoS_2_ when Equation (8) is used [16]. This suggests that accurate calculation of the surface charges that can freely exchange with the external electron reservoir is important for the prediction of grand-canonical barriers, even for the Tafel reaction, in which no interfacial charge transfer occurs. Compared to the canonical result of 0.66 eV, the slightly larger value of the grand-canonical one originates from the surface charge effect.

According to Equation (8), a grand-canonical barrier or reaction energy contains the canonical energy contribution Δ*G*_CE_ and the charge-dependent grand-canonical portion Δ*G*_n,μ_ = −Δ*n*_catal_ × *μ*_e,RHE_. To analyze how surface charge affects the reaction energetics along the reaction route, we plot these two energy terms, together with *n*_total_ and *n*_catal_, in Figure 4d. When moving from the IS to the TS, *n*_total_ increases by only 0.05 e, but *n*_catal_ increases by 0.20 e, implying that electrons keep flowing into the catalyst before the saddle point. The former causes a minor decrease of *G*_CE_ by 0.21 eV, but the latter induces a significant increase of *G*_n,μ_ by 0.92 eV. The total effect is that a barrier of 0.71 eV is generated. Obviously, Δ*G*_n,μ_ destabilizes the system while Δ*G*_CE_ serves a stabilizing factor. Along the whole route, *n*_catal_ remains smaller than *n*_total_, which indicates that *n*_catal_ is a part of *n*_total_ and the former should be used for calculation of reaction energetics. Compared to the canonical case, the grand-canonical FS is more exothermic. This arises from the electron outflow from the catalyst after the saddle point (i.e., *n*_catal_ decreases), which moves Δ*G*_n,μ_ to the negative side. Meanwhile, *n*_total_ keeps increasing, which means that both Δ*G*_CE_ and Δ*G*_n,μ_ become the stabilization factor.

## 3. Methods

### 3.1. DFT Computational Details

Canonical DFT calculations were performed with the Vienna Ab initio Simulation Package (VASP), which uses the Projector Augmented Wave (PAW) method to address electron–nuclei interactions [24,25,26]. The optB86b exchange-correlation function was employed to describe van der Waals (vdW) interactions [27,28,29]. A cutoff energy of 450 eV and a Monkhorst–Pack k-grid of 5 × 3 × 1 were used to obtain energies. Forces on atoms were relaxed with a criterion of 0.03 eV/Å. Reaction transition states were determined using the Climbing Image Nudged Elastic Band (CI-NEB) method [30,31,32,33,34]. Work function drifting along the reaction path was corrected by the charge extrapolation scheme [35]. Zero-point energy changes between two states were included to obtain the free energy difference [36].

Grand-canonical total energy calculations utilized the JDFTx-1.7.0 package with the CANDLE solvent model [24,37]. This package was developed to calculate the joint slab-solution energy (JSSE) for a slab system under a specified voltage [11]. Compared to the canonical results, computational parameters, such as exchange-correlation functions, cutoff energy and convergence criteria, remain the same as those used in VASP.

### 3.2. Canonical Calculation of Hydrogen Adsorption Energy

In a canonical DFT calculation, a catalyst is typically treated as an electrically neutral system. We can calculate the hydrogen adsorption energy by using the following the traditional method [18],

(3) $$∆G_{mH}=∆E_{mH}+∆E_{ZPE}-T∆S$$

(4) $$∆E_{mH}=E_{mH}-E_{\left(m-1\right)H}-\frac{1}{2}E_{H_{2}}$$

where $∆G_{mH}\;$ is the change in free energy for the adsorption of the *m*-th H atom. $E_{mH}$ and $E_{\left(m-1\right)H}$ are the total energies for catalysts with *m* and *m* − 1 H atoms, respectively. $E_{H_{2}}\;$is the total energy of an H_2_ molecule. $∆E_{ZPE}$ is the change in zero-point energy between the adsorbed H atom and the gaseous H_2_. ∆S = 0.5$S_{H_{2}}$ is the entropy change between the adsorbed state and the gaseous state.

### 3.3. Grand-Canonical Calculation of Hydrogen Adsorption Energy

Under electrochemical conditions, however, a catalyst works under a constant external potential (*μ*_e_), which is established by the electron exchange between the catalyst and the external electron reservoir. This means that the catalyst surface charge may change during an electrocatalytic reaction. Therefore, the hydrogen adsorption energy under a given potential could be calculated using the following formula [38]:

(5) $$\begin{array}{c} ∆G_{H}=G_{sol}\left(H^{*Q2}\right)-G_{sol}{(}^{*Q1})-G\left(H^{+}\left(sol\right)\right)-\left(Q1-Q2+1\right)\mu_{e}\\ =G_{sol}\left(H^{*Q2}\right)-G_{sol}{(}^{*Q1})-\frac{G\left(H_{2}\left(g\right)\right)}{2}+\left|e\right|U-\left(Q1-Q2\right)\mu_{e} \end{array}$$

Here, $G_{sol}\left(H^{*Q2}\right)$ and $G_{sol}{(}^{*Q1})$ are the canonical free energies of the catalyst with and without hydrogen adsorption, respectively. $G\left(H^{+}\left(sol\right)\right)$ represents the energy of a proton in the solution, $\left(Q1-Q2+1\right)\mu_{e}$ implicates the surface charge change upon hydrogen adsorption and $\mu_{e}$ is the electron chemical potential, i.e., the Fermi level *E*_F_. *U* is the applied voltage. *Q*1 and *Q*2 are the net surface charges before and after H adsorption, respectively. They should satisfy the following constraint:

(6) $$E_{F}{(}^{*Q1})=E_{F}\left(H^{*Q2}\right)=\mu_{e}$$

### 3.4. Modified GCP-K Method

A grand-canonical potential kinetics (GCP-K) model has been developed in a recent study [13]. This model optimizes the grand-canonical energy (*G*_GCE_) with respect to the net electron number *n*_total_ and geometry coordinate *R* under a given voltage using a quadratic GCP. Specifically speaking, it uses VASP to relax structures for different *n*_total_ and JDFTx with the CANDLE solvation model to obtain the corresponding canonical JSSEs. By fitting the JSSE vs. *n*_total_ curve using a quadratic GCP and locating the minimum, one can determine *n*_total_, *R* and *G*_GCE_ for the system under a specified potential. Then, the reaction barrier (or reaction energy) could be derived from

(7) $$∆G^{‡}=G_{TS/FS,GCE}-G_{IS,GCE}$$

which contains the contribution of *n*_total_. Essentially, *n*_total_ is the result of the electron flow between the catalyst and the external electron reservoir, which establishes the electron chemical potential balance. For an EDL system, however, *n*_total_ represents the total number of electrons distributing not only on the surface, but also on the electrolyte. Only the part residing on the catalyst side (*n*_catal_) can freely exchange with the electron reservoir. Therefore, Equation (7) may overestimate the energy change due to the use of *n*_total_. This is the reason why the GCP-K method fails to reproduce the experimental reaction barrier [16].

Herein, we propose a modified version of the GCP-K method, which can accurately separate *n*_catal_ from *n*_total_ and thus give a more reasonable prediction for reaction kinetics. *n*_catal_ represents the net surface charge that can exchange directly with the external electron reservoir. Under electrochemical conditions, electrons from the external circuit may tunnel through the interface. Therefore, calculation of *n*_catal_ should take into account the water molecules that changes their charges by the same sign as the catalyst does during a reaction. For instance, if the catalyst gains electrons in a reaction, the water molecules that synchronously gain electrons should be treated as the part of the catalyst side. *n*_catal_ can be calculated from the Bader analysis and should be a part of *n*_total_, which will be discussed in the following Section 2.3. Using *n*_catal_, one can rewrite Equation (7) as

(8) $$∆G^{‡}={(G}_{TS/FS,CE}-G_{IS,CE})-\mu_{e,RHE}\left(n_{TS/FS,catal}-n_{IS,catal}\right)$$

The subscript CE denotes the canonical JSSE. $\mu_{e,RHE}$ is the electron chemical potential at a given potential vs. the reversible hydrogen electrode (RHE). For the SHE condition, $\mu_{e,SHE}=-4.66$ eV within the CANDLE solvation model. This formula includes the contribution of *n*_catal_ and, therefore, may give a more reasonable evaluation of reaction energetics. When *n*_catal_ = *n*_total_, Equation (8) becomes Equation (7). In fact, for surfaces without water layers, Equations (7) and (8) give the same result.

Equation (8) divides a grand-canonical barrier (reaction energy) into a charge-dependent grand-canonical portion Δ*G*_n,μ_ = −Δ*n*_catal_ × *μ*_e,RHE_, and a canonical energy component Δ*G*_CE_, facilitating an analysis of the charge-dependent reaction mechanism. The former only correlates with *n*_catal_ at a given potential, whereas the latter is dependent on *n*_total_ (usually a higher *n*_total_ may lead to a lower *G*_CE_) [16]. When electrons flow into the catalyst during a reaction, the second term stabilizes the system whereas the first term destabilizes it. Instead, if there is a net electron outflow during the reaction, the two effects are reversed [16].

## 4. Conclusions

In conclusion, we have developed a modified version of the GCP-K method based on DFT calculations to quantitatively predict reaction kinetics for electrochemical reactions. This method can separate the surface charges that freely exchange with the external electron reservoir and, therefore, can accurately predict reaction barriers. Using Pt(111) as an example, we studied the hydrogen adsorption thermodynamics and the Tafel reaction kinetics. We find that the equilibrium hydrogen adsorption energy under the SHE condition should be around −0.2 eV, rather than 0 eV, if one consider the high density of surface negative charges. Even for the Tafel reaction, in which no charges transfer across the interface, surface charges may lead to a remarkable difference in prediction of reaction barrier. We demonstrate that the net electron inflow into the catalyst during the Tafel reaction leads to a stabilization of canonical energy and a destabilization of the charge-dependent grand-canonical component, inducing a slightly higher barrier (by 0.05 eV). This study provides an effective and practical grand-canonical method to predict reaction kinetics and to analyze the surface charge effect. It is noteworthy that this law is universal for all electrocatalysts because Equation (8) has been derived without specification of the types of materials and reactions. 

## Figures and Tables

**Table 1:** Net electron numbers *n*_total_ for different parts of the interfacial structure (Figure S1) at the grand-canonical initial states and transition states of the Tafel reaction. Δ*n*_total_ indicates the change of electron number *n*_TS,total_−*n*_IS, total_ when moving from the initial state to the transition state. The H_2_O molecules denoted by “*” are selected as a part of the catalyst.

Species	*n*_IS,total_	*n*_TS,total_	Δ*n*_total_
H_2_O-1	7.94	7.90	−0.04
H_2_O-2	7.95	7.95	−0.004
H_2_O-3	8.04	7.98	−0.06
H_2_O-4 *	7.95	8.01	+0.06
H_2_O-5	8.04	8.03	−0.01
H_2_O-6	8.05	8.01	−0.04
H_2_O-7	8.00	7.97	−2.04
H_2_O-8 *	8.03	8.04	+0.01
H_2_O-9 *	7.96	7.99	+0.03
H_2_O-10 *	8.00	8.05	+0.05
Catalyst	390.04	390.08	+0.05
Total	470.0002	470.0001	−2.0001

## Data Availability

The data presented in this study are available on request from the corresponding author.

## Supplementary Materials

The following supporting information can be downloaded at: https://www.mdpi.com/article/10.3390/molecules29081813/s1, Table S1. Corrections from zero-point energy (ΔZPE) and charge extrapolation (ΔCE) for canonical barriers and reaction energies. Figure S1. Structural models of Pt (111)/H_2_O (a) Top and (b) side views of the grand-canonical initial state structure of the Tafel reaction in acid.
