# Supplementary material for: Surface Charge Effects for the Hydrogen Evolution Reaction on Pt(111) Using a Modified Grand-Canonical Potential Kinetics Method
Source: Molecules. 2024 Apr 17;29(8):1813. doi: 10.3390/molecules29081813 (PMC11055056; doi:10.3390/molecules29081813)
Supplement: Supplementary file 1 [file molecules-29-01813-s001.zip › molecules-2898547-supplementary.pdf]

Surface Charge Effects on the Thermodynamics and Kinetics of Hydrogen Evolution Reaction on Pt(111) Using a Modified Grand-Canonical Potential Kinetics Method

Shaoyu Kong,[†,#] Min Ouyang,[†] Yi An,[†] Wei Cao,[†] Xiaobo Chen[†,*]

[†]Guangzhou Key Laboratory of Vacuum Coating Technologies and New Energy Materials, Guangdong Provincial Engineering Technology Research Center of Vacuum Coating Technologies and New Energy Materials, Department of Physics, Jinan University, Guangzhou, Guangdong 510632, China.

[a)]**Author to whom correspondence should be addressed:** txbchen@jnu.edu.cn

Table S1. Corrections from zero-point energy (ΔZPE) and charge extrapolation (ΔCE) for canonical barriers and reaction energies.

		$\Delta E^{‡}$ (eV)	ΔZPE (eV)	ΔCE (eV)	$\Delta G^{‡}$ (eV)
Tafel	reaction barrier	0.79	-0.11	-0.02	0.66
	reaction energy	0.14	-0.02	0.03	0.16

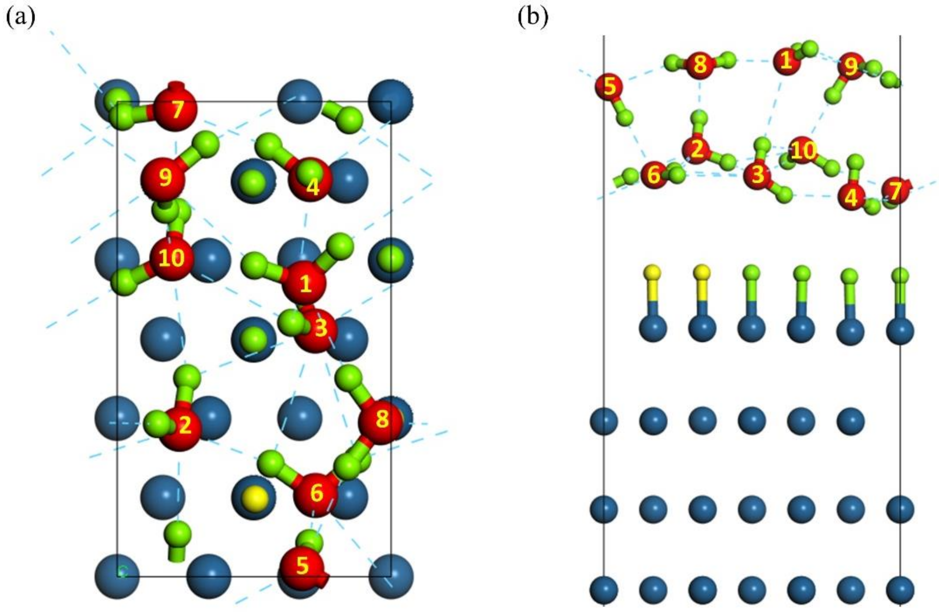

Figure S1. Structural models of Pt (111)/H_2O (a) Top and (b) side views of the grand-canonical initial state structure of the Tafel reaction in acid. There are 10 H_2O molecules marked with numerals in the water layer.
